# COI Metabarcoding as a Novel Approach for Assessing the Honey Bee Source of European Honey

**DOI:** 10.3390/foods14030419

**Published:** 2025-01-27

**Authors:** Mónica Honrado, Andreia Quaresma, Dora Henriques, M. Alice Pinto, Joana S. Amaral

**Affiliations:** 1 CIMO, LA SusTEC, Instituto Politécnico de Bragança, Campus de Santa Apolónia, 5300-253 Bragança, Portugal; monica-honrado@hotmail.com (M.H.); aquaresma7@gmail.com (A.Q.); dorasmh@gmail.com (D.H.); apinto@ipb.pt (M.A.P.); 2 LAQV-REQUIMTE, Department of Chemistry, University of Aveiro, Campus Universitário de Santiago, 3810-193 Aveiro, Portugal

**Keywords:** entomologic origin, *Apis mellifera*, mtDNA, DNA metabarcoding, honey, COI gene

## Abstract

Honey is a widely consumed food product frequently subjected to adulteration, with the mislabeling of its botanical or geographical origin being one of the most common practices. Determining the entomological origin of honey is particularly challenging but of high relevance for ensuring its authenticity, especially for products with protected designation of origin (PDO) labels. This study presents a novel DNA metabarcoding approach targeting a highly informative 406 bp fragment of the cytochrome c oxidase I (COI) gene to differentiate among the three major mitochondrial lineages (A, M, and C) of honey bees (*Apis mellifera* L.) native to Europe. The target region was selected based on the calculated fixation index (F_ST_), which is frequently used in Population Genetics as a measure of differentiation between populations. The approach was validated with 11 honey samples of known entomological origin and applied to 44 commercial honeys from 13 countries. The approach demonstrated high sensitivity, accurately identifying the entomological origin of honey, including samples produced by honey bees of varying ancestries, which could not be resolved by previous methods based on real-time PCR coupled with high-resolution melting (PCR-HRM) analysis. The results demonstrate the effectiveness of COI metabarcoding in verifying honey authenticity and highlight the predominance of C-lineage honey bees in the production of commercial honeys from northwestern Europe. This finding suggests a limited presence of the native M-lineage ancestry, underscoring the need for conservation efforts.

## 1. Introduction

Honey is the sweet substance produced by honey bees (*Apis mellifera* L.) from the nectar of plants or the secretions of the living parts of plants [1]. In the last decades, consumers have become increasingly aware of honey’s health benefits; therefore, its consumption has been steadily growing worldwide [2]. The European Union (EU) is the world’s second-largest honey producer after China, with an annual production of ~228,000 tonnes in 2021, which increased to ~286,000 tonnes in 2022 [3]. However, this amount is not enough to make the EU self-sufficient; therefore, ~190,000 tonnes of honey were imported in 2022 to meet the internal consumption needs [3]. In such a context of high market demand, honey is increasingly a target of economically motivated adulteration and is considered one of the foods most prone to fraud [4,5]. While the addition of sugars/syrups is the most common honey adulteration [6], mislabeling by a false declaration of the botanical or geographical origin is also important.

Consumers frequently favor honeys exhibiting unique organoleptic characteristics primarily determined by their botanical and geographical origins. These honeys typically reach a high market value. This is the case of several European honeys with the label of protected designation of origin (PDO), which are prized for their high quality and unique characteristics associated with their geographical origin. Contrary to the botanical origin, which has traditionally been assessed by melissopalynology, the geographical origin of honey is difficult to determine, and it is frequently inferred from the pollen composition of honey based on the characteristic flora signature of the corresponding regions [7]. However, this indirect approach is inconclusive when the flora signature is unknown, when different regions share similar floras, or when the honey is derived from nectar-secreting plants that are poor pollen producers. Establishing the geographical origin of honey remains a challenging endeavor that may require an orthogonal approach combining data from different techniques. Therefore, several works have focused on different approaches, such as the use of multielemental profiles and isotopic signatures [8,9,10] and DNA analysis to establish the entomological origin [11,12,13,14,15,16,17,18]. While the former approach infers the geographical origin from the correlation between the elemental and/or isotopic composition of the soil and water of the production site and that of the honey, the latter considers the native distribution of *Apis mellifera* subspecies.

The *A. mellifera* diversity native to Europe is clustered into three evolutionary lineages: Western European (M), Eastern European (C), and African (A), a pattern concurrently supported by morphology [19], mitochondrial (mt) DNA [20], and single-nucleotide polymorphisms (SNPs) [21]. Despite the changes in the last decades in the natural distribution of the European honey bee subspecies due to the large-scale movement and introduction of exotic queens in many countries [22], the entomological origin of honey remains a valuable parameter to ascertain the honey origin, particularly PDO honey [11,17]. PDO food has a strong link to the place where it is made; therefore, in theory, PDO honey should be produced by native honey bee subspecies. In practice, this is acknowledged by some European PDO honeys, which introduce specifications on the honey bee subspecies on the EU geographical indications register [23]. A good example is the most recently approved Croatian honey “Goranski medun”, which should be produced by the Carniolan honey bee [24].

Up until now, most methodologies that have been put forward to distinguish honeys produced by different subspecies are based on DNA analysis [12,13,14,15], with only a few works attempting to differentiate the three lineages occurring in Europe [11,16,17,18]. These works have proposed the employment of the qualitative polymerase chain reaction (PCR) [17], real-time PCR coupled with high-resolution melting (HRM) analysis [11,16], or a multi-step approach based on qualitative PCR followed by Sanger sequencing [18]. Despite these advances, next-generation sequencing (NGS) remains underexploited for this purpose. In recent years, NGS has played a crucial role in species authentication as it can detect species mixtures or adulteration in food samples [25,26,27]. Its ability to simultaneously generate massive amounts of different DNA sequences and the possibility of accurate species identification through the comparison with genomic databases makes NGS a well-suited tool for identifying ingredients in complex foods [28]. This is often achieved by DNA metabarcoding through the generation of DNA sequences from one or more well-characterized genomic regions that are present in all members of a taxonomic group of interest but possess sufficient sequence variability to allow the identification of the organism of origin (barcode regions).

DNA metabarcoding has numerous applications, including monitoring biodiversity changes over time, detecting the presence of non-native or invasive species, and characterizing complex organismal communities in various habitats, among others [29,30]. More recently, DNA metabarcoding has also been gaining popularity in establishing the botanical origin of honey as an alternative to melissopalynology [31,32,33,34,35,36,37,38,39]. So far, only a few works have exploited the potential of DNA metabarcoding for the entomological authentication of honey. Prosser and Hebert suggested the use of this approach to establish the botanical and entomological origin of six samples of honey produced by *A. mellifera* and one by *Melipona beecheii* [14]. However, to the best of our knowledge, no attempts have been made to differentiate honeys produced by distinct honey bee subspecies. Herein, we propose a DNA metabarcoding approach based on the amplification of an informative short fragment of the cytochrome c oxidase I (COI) gene to identify honeys produced by the three evolutionary lineages present in Europe (A, M, and C). This NGS-based approach was applied to 11 samples of known entomological origin and 44 commercial honeys from different European countries. The results were compared with data previously obtained by real-time PCR coupled with HRM analysis.

## 2. Materials and Methods

### 2.1. Samples

Eleven authentic honey samples with known entomological origin were examined (Table 1). Of these, five were collected by beekeepers in Portugal, four in Italy and two in Spain, corresponding to the mitochondrial A-lineage *A. m. iberiensis*, C-lineage *A. m. ligustica* and M-lineage *A. m. iberiensis*. In addition, a total of 44 commercial honey samples produced in different countries were purchased on e-commerce international markets and at local Portuguese stores, namely, Portugal (*n* = 19), Spain (*n* = 4), Italy (*n* = 2), France (*n* = 4), Norway (*n* = 1), Germany (*n* = 3), Sweden (*n* = 1), Estonia (*n* = 1), Finland (*n* = 1), Slovenia (*n* = 4), Latvia (*n* = 2), Lithuania (*n* = 1), and New Zealand (*n* = 1). The samples were of different botanical origins and included multi- and mono-floral honeys (Table 1).

### 2.2. DNA Extraction from Honey

For each honey sample, a pre-treatment step was carried out to eliminate possible interferents, such as sugars and polyphenols, as previously described by Honrado et al. [11]. Twenty-five grams of honey were divided into two 85 mL centrifuge tubes (12.5 g per tube) and mixed with 40 mL ultrapure water in each tube. The mixtures were stirred and incubated at 56 °C for 15 min, and centrifuged at 16,000× *g* for 15 min at 4 °C, and the supernatants were discarded. The resulting pellets were resuspended in 40 mL ultrapure water and centrifuged under the same conditions. After discarding the supernatants, the pellets were resuspended in approximately 1 mL ultrapure water, transferred to 2 mL reaction tubes, and centrifuged once more. The pellets were resuspended in 1 mL ultrapure water, centrifuged again, and frozen overnight at −20 °C. They were then stored at −80 °C for 3 h before being heated at 56 °C for 10 min in a thermoblock to induce cell lysis via thermal shock. Following centrifugation under the same conditions, the pellets were washed with ultrapure water, concentrated by centrifugation, and stored at −20 °C until DNA extraction. DNA was extracted using a NucleoSpin^®^ Plant II kit (Macherey-Nagel, Düren, Germany), according to the manufacturer’s instructions with minor modifications as described by [40]. The nucleospin lysis buffer (PL1) was added to the samples, which were then transferred to 2.0 mL screwcap tubes containing a mixture of zirconia beads of varying sizes. The samples were homogenized using a Precellys 24 tissue homogenizer (Bertin Instruments, Montigny-le-Bretonneux, France) at 6200 rpm for three 5 s cycles. After homogenization, the samples were incubated for 2 h, and DNA extraction was completed according to the manufacturer’s instructions.

The quality and yield of the DNA extracts were assessed by UV spectrophotometry using a SPECTROstar^®^ Nano microplate reader (BMG Labtech, Offenburg, Germany) with an LVis plate accessory. To estimate DNA content and purity, the absorbance was measured at 260, 280, and 230 nm using the Multi-user Reader Control and MARS Data Analysis Software version 5.70 R2 (LVis, BMG Labtech, Offenburg, Germany). The DNA extracts were kept at −20 °C until further analysis and diluted to 10 ng/μL before PCR.

### 2.3. Selection of the Informative Region

The informative region for downstream DNA metabarcoding analysis was identified from 95 mitochondrial genomes (mitogenomes), representing four honey bee subspecies (80 *A. m. iberiensis*, eight *A. m. mellifera*, four *A. m. ligustica*, and three *A. m. carnica*) and three lineages (59 A, 38 M, and 24 C) obtained by Whole Genome Sequencing (WGS) on an Illumina HiSeq 2500 platform (see further details in Henriques et al. [41]). A total of 645 SNPs distributed across all genes were identified from the 16,343 bp reference mitochondrial genome [41]. The genetic differentiation between lineages was determined using the fixation index (F_ST_) calculated by VCFtools—v0.1.12a [42]—from 500 bp sliding windows. The size of the sliding window was selected based on the size of the sequence reads generated by the Illumina MiSeq platform using the Illumina 2 × 250 cycles v2 nano chemistry (see below).

### 2.4. DNA Metabarcoding

**Figure 1:**
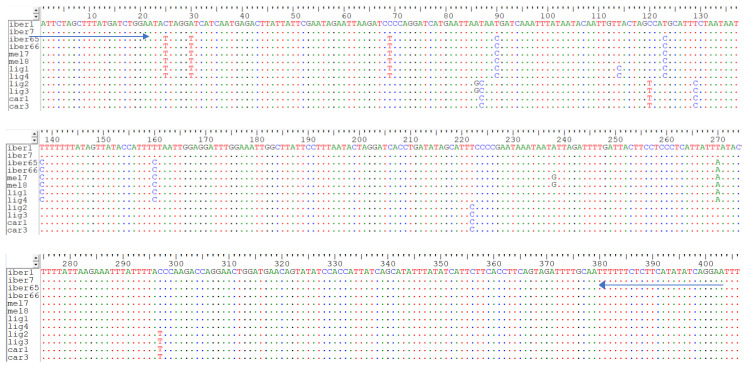
Alignment of the COI sequence corresponding to the 406 bp fragment amplified by the newly designed primers COI_NGS-F/COI_NGS-R (identified by the arrows) of representative sequences of lineage A *A. m. iberiensis* (iber1 and iber7), lineage M *A. m. iberiensis* (iber65 and iber66), lineage *A. m. mellifera* (mel7 and mel8), lineage M *A. m. ligustica* (lig1 and lig4), lineage C *A. m. ligustica* (lig2 and lig3), and lineage *A. m. carnica* (car1 and car3).

DNA metabarcoding was performed using a two-step indexing approach and a set of newly designed primers targeting a 406 bp fragment of the COI gene containing 11 SNPs identified from the mitogenomes as the most informative for differentiating the three lineages (A, M, and C) (Figure 1). The two-step indexing approach used in this work allows for a streamlined process that separates the target amplification from the indexing and adapter incorporation stage, minimizing contamination risks and increasing protocol flexibility. The first PCR was carried out in triplicate for each sample using the primers COI_NGS, which were modified to contain the overhang adapters proposed by Illumina [43], namely, COI_NGS-F 5′-*TCGTCGGCAGCGTCAGATGTGTATAAGAGACAG***ATTCTAGCTTTATGATCTGGAA**-3′ and COI_NGS-R 5′-*GTCTCGTGGGCTCGGAGATGTGTATAAGAGACAG***AAATTCCTGATATATGAAGAGAA**-3′ (the newly designed primers are highlighted in bold, and the nucleotides marked in italics represent the overhangs proposed by Illumina [43] required for NGS to allow the incorporation of the unique indexes and the P5 and P7 adaptors from Illumina into each sample). The first PCR was performed in a total volume of 10 μL, containing 0.5 μL of each primer at 10 μM, 5 μL of Q5 High-Fidelity 2X Master Mix (New England Biolabs, Ipswich, MA, USA), and 1 μL of DNA at 10 ng/μL. Thermal cycling conditions were 98 °C for 3 min, 35 cycles of 98 °C for 10 s, 55 °C for 30 s, and 62 °C for 40 s, as well as a final extension of 62 °C for 2 min. The amplicons were then sent to the Centre for Molecular Analyses (CTM), the laboratory of the Research Centre in Biodiversity and Genetic Resources (CIBIO; Vairão, Portugal). There, the amplicons were purified using 0.8× reversible immobilization paramagnetic beads (Agencourt AMPure XP) per microliter of PCR product. The purified amplicons were then subjected to a second PCR to incorporate custom-made unique indexes and the P5 and P7 adaptors from Illumina (San Diego, CA, USA). The custom-made indexes were adapted from Kircher et al. [44] and Gansauge and Meyer [45]. These allow the pooling of a maximum of 1920 samples and are the indexes preferentially used at CTM, following the protocol described by Paupério et al. [46]. The second PCR reaction was performed in a 10 μL total volume containing 0.5 μL of each oligonucleotide at 1 μM, 5 μL of KAPA HiFi HotStart ReadyMixPCR Kit (Kapa Biosystems, Wilmington, MA, USA), and 2 μL of 1:10 dilution of the purified amplicons. Thermal cycling conditions were 95 °C for 3 min, followed by 10 cycles of 95 °C for 30 s, 55 °C for 30 s, 72 °C for 30 s, and a final extension of 72 °C for 5 min. The indexed amplicons were then purified (using the purification reaction as before), quantified in the Epoch Microplate Spectrophotometer (Bio Tek Instruments, Winooski, VA, USA), normalized to a final concentration of 10 nM, and pooled. For each pool, the amplicon size distribution was determined on a TapeStation 2200 using the HS D1000 kit (Agilent Technologies, Santa Clara, CA, USA) for quality control purposes, and the quantification was performed using a SYBR green quantitative PCR assay with the KAPA Library Quantification kit (Kapa Biosystems). The pools were combined equimolarly into one single sequencing library containing all samples. The sequencing library was diluted to 2 nM, spiked with 10% Illumina-generated PhiX control library, and then sequenced on the Illumina MiSeq using the 2 × 250 cycles v2 nano chemistry, according to the manufacturer’s instructions.

### 2.5. Sanger Sequencing

Given the paucity of available published sequences of A. m. carnica, and to supplement the 95 sequences obtained by WGS and previously published [41], the DNA extracted from the thorax of 19 *A. m. carnica* individuals was amplified using the primers COI_NGS-F/COI_NGS-R and the PCR conditions described in Section 2.4. These individuals were collected in the native range of *A. m. carnica* in Croatia and Serbia, and their ancestries were previously confirmed by ADMIXTURE [47] analysis of whole genomes (MAP, unpublished data). The PCR products were sent to STABVIDA (Lisbon, Portugal) for purification and Sanger sequencing in both directions. The sequences were analyzed and aligned by BioEdit v7.2.5 (Ibis Bio-sciences, Carlsbad, CA, USA) and MEGA-X [48].

### 2.6. Bioinformatics

Based on the unique indexes incorporated in each sample in the second PCR, pools were de-multiplexed in the BaseSpace Sequence Hub. Raw sequence reads (fastq files) were processed using VSEARCH v2.15.2 [49]. Reads R1 and R2 were merged using *fastq_mergepairs*, low-quality reads (length 550 bp, ambiguous base pairs) were discarded using the *fastq_filter*, and chimeras were detected and discarded using *uchime3_denovo*. After these filtration steps, sequence reads were classified directly to the subspecies level using *usearch_global* with a sequence similarity threshold of 99%. The COI reference database used in the taxonomic classification was built from the 95 aforementioned mitogenomes [41]. Two files were produced by the taxonomic classification: a community matrix table, where columns represent samples and rows represent subspecies, and a file with the mtDNA lineage (A, M, or C) of each subspecies. The files were imported into R-Studio v1.2.5033 [50] using the package Phyloseq v1.27.6 [51], and the subspecies relative abundances detected in each sample were estimated after discarding taxa with fewer than 10 reads.

## 3. Results and Discussion

### 3.1. Target Gene Selection and Method Development

To select the most informative region for differentiating lineages A, C, and M, the mitogenomes of the 95 honey bees obtained by Henriques et al. [41] were used. For that purpose, the mean fixation index (F_ST_) was calculated for each 500 bp sliding window run across the complete mitogenomes. F_ST_ is frequently used in Population Genetics as a measure of differentiation between populations [52]. Analysis of the sliding windows showed the highest mean F_ST_ (0.94) for the COI gene, corresponding to the region located between 1794 bp and 2371 bp in the reference mitogenome (NC_001566.1). Therefore, new primers were manually designed to amplify a fragment in this highly informative region while showing adequate characteristics regarding the possible formation of primer–dimer or hairpins. The new primers amplify a 406 bp fragment located between positions 1848 bp and 2253 bp in the COI gene. For complex matrices, such as honey, the amplification of short fragments (up to 250 bp) is typically favored due to the high probability of DNA degradation. Previous works relied on the use of smaller fragments, including <170 bp for assays based on the PCR amplification of the tRNA^leu^-COX2 region, followed by gel electrophoresis [17] or sequencing [18], and <151 bp for assays based on high-resolution melting (HRM) analysis [4,11]. Particularly, when using HRM, amplicons should be kept under 300 bp as the length influences the sensitivity of the analysis, with longer amplicons resulting in smaller differences in the melting curves [53]. Based on the results of the F_ST_ analysis, in this work, we chose to target a longer fragment to cover a larger number of SNPs, enabling a more accurate differentiation of the three mitochondrial lineages (A, C, and M) present in Europe. The selected 406 bp fragment included 13 SNPs (Figure 1), of which 11 presented a calculated F_ST_ = 1. The selected amplicon length allowed us to capture as much variation as possible to resolve the lineages within the recommended range (~450 bp) for the Illumina 2 × 250 cycles v2 nano chemistry. Subsequently, the designed primers, without the overhangs, were tested using different DNA extracts obtained from honey samples, evidencing positive PCR amplification and confirming their effectiveness. After optimizing the annealing temperature of the PCR, a small set of amplicons now amplified using the primers with the overhangs (see Section 2 for details) were Sanger-sequenced to confirm the specificity and quality of the amplicons.

### 3.2. Application of DNA Metabarcoding to Identify the Entomological Origin of Honey

The proposed metabarcoding approach, consisting of the amplification of an informative 406 bp fragment of the COI gene followed by NGS, was then applied in 55 honey samples, including 11 of known entomological origin and 44 commercial honeys. The Illumina MiSeq run produced 342,069 paired-end raw reads for the multiplexed library. After all quality filtering steps, the final dataset consisted of 89,738 paired-end reads. The sequencing depth per sample ranged from 37 to 5670 reads, with a mean of 1559 reads per sample. To overcome potential biases from amplification or sequencing errors, SNPs were deemed reliable only if, after quality filtering, they were identified in at least 10 different reads assigned to the same subspecies [14,54].

To assess the reliability of the COI metabarcoding approach in determining the entomological origin of honey, 11 samples of known entomological origin supplied by beekeepers from Portugal (*n* = 5), Spain (*n* = 2), and Italy (*n* = 4) were tested (Table 1). The yield and purity of the 11 DNA extracts ranged from 6.1 to 239.3 ng/μL and from 1.3 to 2.0, respectively. These 11 honey samples were tested previously by real-time PCR coupled with high-resolution melting analysis (PCR-HRM) (results disclosed in [11]). The comparison of the two independent methods is summarized in Table 1, which shows the metabarcoding results expressed as relative abundances.

As shown in Table 1, all sequencing reads (100%) obtained for the Portuguese honey samples matched the A-lineage *A. m. iberiensis*, aligning with the beekeeper’s information and the predominant African maternal lineage endemic to Portugal. These NGS results are consistent with previous findings obtained with the PCR-HRM method [11]. Similarly, the sequencing reads generated from the authentic honey samples from Spain and Italy showed 100% correspondence to the M-lineage and C-lineage, respectively, further validating the efficacy of the newly developed DNA metabarcoding approach for entomological authentication.

Therefore, the validated COI metabarcoding approach was applied to 44 commercial honeys acquired from different geographical origins, including 12 European countries and New Zealand. The yield and purity of the honey DNA extracts of the commercial samples ranged from 5.3 to 1090.0 ng/μL and from 1.3 to 2.0, respectively. These honeys were previously tested by the PCR-HRM approach [11], and a comparison of both approaches is summarized in Table 2. Apart from sample H35, all of the other honey samples exhibited consistent results between COI metabarcoding and PCR-HRM, including those previously identified as produced by a single ancestry and those classified as “not determined” as they consisted of mixtures of different lineages.

Regarding the honey samples previously classified by PCR-HRM as being produced by a single lineage (H2–H9, H16–H21, H24, H27, H29, H30, H31, and H33) identical results were obtained by NGS, corroborating the previous classification. For most of the commercial honey samples from different regions of Portugal, namely, H2–H9 (Alentejo), H16–H19 (Lousã), H1 (Terceira, Azores), and H13 (São Miguel, Azores), all sequencing reads (100%) aligned with lineage A, as expected from the prevailing African maternal ancestry of the Portuguese honey bee populations [55,56] and consistent with the PCR-HRM findings [11]. Two honeys from Spain (H20 and H21) were also consistent with the previous study [11] as they were shown to be produced exclusively by honey bees of A-lineage ancestry. Similarly, the manuka honey from New Zealand (sample H24) and the three Corse PDO honeys from France (H27, H29, and H30) were classified as being produced by C- and M-lineages, respectively, which is also in good agreement with both their provenience and previous PCR-HRM results (Table 2). This result is particularly relevant for the French PDO honeys since it authenticates their entomological origin (and indirectly the geographic provenience). Here, according to the product specification registered in the EU eAmbrosia database [23], Corse PDO honey should be produced by honey bees of the Corse ecotype corresponding to *A. m. mellifera* (M-lineage). Results obtained for samples H31 and H33 from northern Germany and H34 from Sweden were also consistent with the introduced dominant subspecies [57,58] and the previous PCR-HRM identification [11] as they were all classified as belonging to the C-lineage. Four additional honey samples (H11, H15, H26, and H28) successfully examined by PCR-HRM [11] generated a very low number of reads (5, 15, 14, and 3, respectively) and, therefore, were not identified (Table 2). This can possibly be due to DNA degradation and/or low initial DNA template concentrations, consistent with the high Ct values observed during the PCR-HRM analysis, particularly for samples H11 and H15, which were the ones with the highest Ct (35.2 and 34.7, respectively). DNA degradation is a strongly favored explanation for the very low level of amplification observed in these four honeys because the newly designed COI primer pair targets a larger fragment than the one used in the PCR-HRM approach (406 bp versus 150 bp).

The remaining samples were previously classified by PCR-HRM as “not determined” as they did not match any reference cluster, suggesting that they corresponded to honey blends produced by honey bees of varying ancestries [11]. This was not a surprising result as the honey bottled by beekeepers is typically a mixture obtained from several colonies or even apiaries [59]. Moreover, commercial honeys may correspond to blends produced by different beekeepers, which may use honey bees of varying ancestries. For these samples, the COI metabarcoding results confirmed the presence of multiple mtDNA ancestries. This is a major advantage of this approach as NGS allows the simultaneous sequencing of different amplicons in a mixture, surpassing the limitations of PCR-HRM. In this regard, the COI metabarcoding results of H10 and H12, both samples from the Bragança region (northeastern Portugal), further confirmed that they were a mixture of honeys produced by honey bees of A- and M-lineages’ maternal ancestries. This result corroborates the geographical origin of these two honeys as A- and M-lineage mitotypes are sympatric within the native distribution of *A. m. iberiensis*, particularly in this cross-border area between Portugal and Spain [55], with beekeepers frequently having both maternal ancestries in their apiaries. Samples H22 and H23 from the Alicante region (Spain) also contained a mixture of A- and M-lineages, with a prevalence of the latter. Despite not being a quantitative technique, the percentage of the sequencing reads retrieved from each sample can provide a glimpse of the relative amounts of honeys originating from different ancestries, which is an additional advantage of the COI metabarcoding approach developed here as compared to other molecular methods. These results are also in line with data reported by Chávez et al. [56], who carried out a comprehensive survey on the maternal DNA variation across three north–south transects in the Iberian Peninsula, showing the co-occurrence of A- and M-lineages in the Alicante region. Finally, mtDNA belonging to the A-lineage was also identified in the incense honey (H14) from the Azorean island of Faial (Portugal), together with the C-lineage. The presence of these two lineages in sample H14 was also detected in the real-time PCR product sequenced by the Sanger method [11]. The COI metabarcoding approach not only confirms this previous result but also indicates the predominance of C-lineage in H14, as revealed by the higher percentage of sequencing reads aligning with this lineage. Notably, the relative abundances of lineages C and A in the H14 honey sample closely match their frequencies in the honey bee population of Faial (75% and 25% of lineage C and A, respectively) [60]. All the remaining honey samples (H25 and H31–H44) were classified as belonging to lineage C. These samples correspond to commercial honeys produced in Italy, Slovenia, Germany, Latvia, Lithuania, Sweden, Finland, and Norway (Table 2). Interestingly, except for Italy and Slovenia, all these countries were once occupied by the dark European honey bee, *A. m. mellifera*. According to Ruttner, *A. m. mellifera* was native to a large territory expanding from the Pyrenees to Scandinavia and from the British islands to the Urals [19]. However, over the last 150 years, other subspecies, mainly the C-lineage *A. m. carnica* and *A. m. ligustica*, have been massively introduced in large tracts of western and Central Europe [22,50,57,61]. As a result, the natural range of *A. m. mellifera* has been significantly reduced [60], with reports on its virtual replacement in countries like Germany, where *A. m. carnica* is largely dominating [62], or in Scandinavia, where *A. m. ligustica* has been favored by many beekeepers [57,63,64]. The recognition of the threat posed to *A. m. mellifera* by large-scale importations of C-lineage honey bees boosted conservation efforts in different countries [22,60,61,64,65]. Nonetheless, exotic subspecies seem to be still favored in the *A. m. mellifera* distributional range, as suggested by the results obtained for the commercial honey analyzed in this work. Remarkably, the honeys from Germany, Latvia, Lithuania, Norway, Finland, and Sweden were all assigned to C-lineage ancestries. The exception was a single sample (H35) from Estonia, which contained a mixture of C- and M-lineage mtDNA, suggesting the existence of small refuges of *A. m. mellifera*, as reported elsewhere [22,58,63]. Interestingly, the earlier screening of the sample H35 by PCR-HRM detected only *A. m. carnica* mtDNA (Table 2). In the PCR-HRM analysis, this sample most likely clustered with the *A. m. carnica* reference due to the high abundance of C-derived DNA. The detection of a low proportion of M-derived mtDNA by the COI metabarcoding approach underscores its superior sensitivity compared to PCR-HRM, enabling the identification of entomological origin when DNA from multiple lineages is present, even in low amounts.

The enhanced sensitivity of the COI metabarcoding approach was further revealed by the analysis of the samples H32 and H37–H44, which were previously classified as “not determined” by the PCR-HRM approach, consistent with the presence of a DNA mixture [11]. In contrast, they were classified as being of C-lineage by the COI metabarcoding approach (Table 2). However, they contained a mixture of distinct C-derived sequencing reads, explaining the “not determined” classification. Notably, a detailed analysis of the reads showed their match to two types of COI sequences, which differed at position 86 (corresponding to position 1933 in the reference genome NC 001566) by a substitution of adenine for guanine. The analysis of the seven C-lineage mitogenomes, represented in the 95 whole-genome collection previously sequenced by Henriques et al. [41], revealed that the sequences containing adenine corresponded to *A. m. carnica,* whereas those containing guanine corresponded to *A. m. ligustica.* Due to the low number of individuals, to further investigate whether this A/G SNP could differentiate the two C-lineage subspecies, additional information was retrieved from Genbank (134 *A. m. ligustica* and 5 of *A. m. carnica* mitogenomes, plus the COI gene of 1 *A. m. ligustica* and 3 *A. m. carnica* (see accession numbers in Supplementary Materials). Moreover, because the number of *A. m. carnica* sequences was still low, the COI gene was obtained from an additional 19 individuals of *A. m. carnica* (from Croatia and Serbia) by Sanger sequencing. The results showed that the SNP was not diagnostic since it could not accurately discriminate the two C-lineage subspecies. Nevertheless, *A. m. ligustica* showed a very high prevalence of guanine (131 in 137; 95.6%), whereas *A. m. carnica* showed a higher variability since only 76.7% (23 in 30) individuals harbored the alternative adenine nucleotide. In summary, although the A/G SNP is not diagnostic, it explains the “not determined” classification previously obtained for the samples H32 and H37-H44 by the PCR-HRM approach (Table 2).

## 4. Conclusions

In this study, a DNA metabarcoding approach targeting an informative COI region is proposed to evaluate the entomological origin of honey. To select this region, analytical tools from Population Genetics were applied for the first time. The target COI fragment contained 11 SNPs with F_ST_ = 1, which evidences highly differentiated populations, enabling the accurate discrimination of the three major mitochondrial ancestries of European honey bees (lineages A, M, and C). To demonstrate its effectiveness, the proposed approach was first applied to a set of honey samples with known origin and then to commercial honey samples from 13 countries. The results were compared with those previously obtained for the same samples using a real-time PCR approach coupled with high-resolution melting analysis (PCR-HRM). The two approaches showed similar performance in the honey samples with a single entomological origin. However, for samples containing mixtures of honey produced by honey bees of varying ancestries, which could not be classified by the PCR-HRM, the COI metabarcoding approach successfully identified the different sequences and revealed their different entomological origin. Additionally, an estimate of the relative amounts of honey produced by different lineages in mixed honey samples was obtained from the total number of sequencing reads.

The COI metabarcoding approach proved to reliably identify DNA from different mitochondrial lineages, enabling not only the verification of the entomological origin of the honeys under analysis but also the confirmation of their geographical origin. This capability is especially important for honeys claimed to be produced by native honey bees, here illustrated by the French PDO honey from Corsica, which were produced by the native *A. m. mellifera*, consistent with the label.

Finally, our results showed that the honeys from northwestern European countries were mainly produced by honey bees of C-lineage ancestry, with the M-lineage characteristic of the native *A. m. mellifera* found only in one honey sample from Estonia and in low amounts (estimated in 6%). These results are congruent with a contemporary history of the large-scale importation of C-lineage honey bees and demonstrate the need to strengthen conservation efforts to protect the native diversity shaped by thousands of years of natural selection.

## Figures and Tables

**Table 1:** Comparison of results obtained by COI metabarcoding (this study) and real-time PCR coupled with HRM (PCR-HRM; [11]) approaches applied to authentic honey samples.

Honey Sample ^a^	Geographical Origin	Botanical Origin	Entomological Origin	PCR-HRM ^b^ [11]	COI Metabarcoding (%) ^c^		
					Lineage A	Lineage C	Lineage M
1	Portugal, Alentejo	Multifloral	*A. m. iberiensis* (A)	A	100	0	0
2	Portugal, Alentejo	Multifloral	*A. m. iberiensis* (A)	A	100	0	0
3	Portugal, Alentejo	Multifloral	*A. m. iberiensis* (A)	A	100	0	0
4	Portugal, Alentejo	Multifloral	*A. m. iberiensis* (A)	A	100	0	0
5	Portugal, Alentejo	Multifloral	*A. m. iberiensis* (A)	A	100	0	0
6	Spain, Tarragona	Multifloral	*A. m.iberiensis* (M)	M	0	0	100
7	Spain, Tarragona	Multifloral	*A. m.iberiensis* (M)	M	0	0	100
8	Italy, Bologna	Multifloral	*A. m. ligustica* (C)	C (*A. m. ligustica*)	0	10	0
9	Italy, Bologna	Multifloral	*A. m. ligustica* (C)	C (*A. m. ligustica*)	0	100	0
10	Italy, Bologna	Acacia	*A. m. ligustica* (C)	C (*A. m. ligustica*)	0	100	0
11	Italy, Bologna	Lime	*A. m. ligustica* (C)	C (*A. m. ligustica*)	0	100	0

^a^ Samples obtained from apiaries studied in previous works [11]; ^b^ results of PCR-HRM were retrieved from [11] and are presented for comparison with metabarcoding results; ^c^ results correspond to the relative abundances calculated from the sequencing reads (%).

**Table 2:** Comparison between COI metabarcoding (this work) and PCR-HRM [11] approaches applied to the analysis of commercial honeys from different countries.

Honey Sample	Geographical Origin	Botanical Origin	PCR-HRM ^a^	COI Metabarcoding (%) ^b^		
			Lineage ^a^	Lineage A	Lineage C (SNP A/G)	Lineage M
H1	Portugal, Terceira, Azores	Multifloral	A	100	0	0
H2	Portugal, Alentejo	Multifloral	A	100	0	0
H3	Portugal, Alentejo	Multifloral	A	100	0	0
H4	Portugal, Alentejo	Multifloral	A	100	0	0
H5	Portugal, Alentejo	Multifloral	A	100	0	0
H6	Portugal, Alentejo	Multifloral	A	100	0	0
H7	Portugal, Alentejo	Multifloral	A	100	0	0
H8	Portugal, Alentejo	Multifloral	A	100	0	0
H9	Portugal, Alentejo	Multifloral	A	100	0	0
H10	Portugal, Bragança	Multifloral	n.d.	22	0	78
H11	Portugal, Bragança (PDO)	Multifloral	n.d.	l.n.r ^c^	l.n.r ^c^	l.n.r ^c^
H12	Portugal, Bragança (PDO)	Rosemary	n.d.	47	0	53
H13	Portugal, S. Miguel, Azores(PDO)	Incense	A	100	0	0
H14	Portugal, Faial, Azores	Incense	n.d.	32	68 (100/0)	0
H15	Portugal, S. Miguel (Azores)	Incense	n.d.	l.n.r ^c^	l.n.r ^c^	l.n.r ^c^
H16	Portugal, Lousã	Orange	A	100	0	0
H17	Portugal, Lousã	Heather	A	100	0	0
H18	Portugal, Lousã	Eucalyptus	A	100	0	0
H19	Portugal, Lousã (PDO)	Heather	A	100	0	0
H20	Spain, Alicante	Multifloral	A	100	0	0
H21	Spain, Alicante	Avocato	A	100	0	0
H22	Spain, Alicante	Carob tree	n.d.	27	0	73
H23	Spain, Alicante	Medlar	n.d.	32	0	68
H24	New Zealand	Manuka	C (*A. m. ligustica*)	0	100 (100/0)	0
H25	Italy	Multifloral	n.d.	0	100 (29/71)	0
H26	Italy, Belluno	Multifloral	n.d.	l.n.r ^c^	l.n.r ^c^	l.n.r ^c^
H27	France, Corse (PDO)	Clementine flowers	M	0	0	100
H28	France, Corse (PDO)	Autumn maquis of ivy flowers	M	l.n.r ^c^	l.n.r ^c^	l.n.r ^c^
H29	France, Corse (PDO)	Spring maquis	M	0	0	100
H30	France, Corse (PDO)	Autumn maquis	M	0	0	100
H31	Germany, north	Dandelion	C (*A. m. ligustica*)	0	100 (100/0)	0
H32	Germany, north	Acacia	n.d.	0	100 (56/44)	0
H33	Germany, north	Summer flowers	C (*A. m. carnica*)	0	100 (0/100)	0
H34	Sweden	Raspberry	C (*A. m. carnica*)	0	100 (0/100)	0
H35	Estonia	Wild fruits	C (*A. m. carnica*)	0	94 (0/94)	6
H36	Slovenia	Chestnut	C (*A. m. carnica*)	0	100 (12/88)	0
H37	Slovenia	Acacia	n.d.	0	100 (54/46)	0
H38	Slovenia	Fir	n.d.	0	100 (18/82)	0
H39	Slovenia	Linden	n.d.	0	100 (35/65)	0
H40	Latvia	Buckwheat	n.d.	0	100 (15/85)	0
H41	Latvia	Heather	n.d.	0	100 (65/35)	0
H42	Finland	Summer flowers	n.d.	0	100 (89/11)	0
H43	Lithuania	Wild	n.d.	0	100 (19/81)	0
H44	Norway	Mountain flowers	n.d.	0	100 (21/79)	0

^a^ Results of PCR-HRM were retrieved from Honrado et al. [11] and are presented for comparison with COI metabarcoding results; n.d.—not determined (did not group with any reference cluster); ^b^ results correspond to the relative abundances calculated from the sequencing reads (%).^c^ results are not shown due to the low number of sequencing reads (l.n.r) generated by the MiSeq run (<10 in each lineage).

## Data Availability

The original contributions presented in this study are included in the article/Supplementary Materials. Further inquiries can be directed to the corresponding author.

## Supplementary Materials

The following supporting information can be downloaded at: https://www.mdpi.com/article/10.3390/foods14030419/s1, Supplementary File S1. Accession numbers of GenBank sequences used in the study.
