# Supplementary material for: COI Metabarcoding as a Novel Approach for Assessing the Honey Bee Source of European Honey
Source: Foods. 2025 Jan 27;14(3):419. doi: 10.3390/foods14030419 (PMC11816727; doi:10.3390/foods14030419)
Supplement: Supplementary file 1 [file foods-14-00419-s001.zip › foods-3380032-supplementary.pdf]

Article

COI Metabarcoding as a Novel Approach for Assessing the Honey Bee Source of European Honey

Mónica Honrado [1,2,3], Andreia Quaresma[1,2], Dora Henriques[1,2], M. Alice Pinto[1,2], Joana S. Amaral[1,2,*]

[1] CIMO, LA SusTEC, Instituto Politécnico de Bragança, Campus de Santa Apolónia, 5300-253 Bragança, Portugal; monica-honrado@hotmail.com (M.H.); aquaresma7@gmail.com (A.Q.); dorasmh@gmail.com (D.H.); apinto@ipb.pt (M.A.P.)

[2] LAQV-REQUIMTE, Department of Chemistry, University of Aveiro, Campus Universitário de Santiago, 3810-193 Aveiro, Portugal

* Correspondence: jamaral@ipb.pt

Supplementary material

In this work, the following sequences retrieved from GenBank were used:

Full mitochondrial genomes of *A. mellifera ligustica*: NC_001566.1, OM203348.1, OM203347.1, OM203346.1, OM203345.1, OM203344.1, OM203343.1, OM203342.1, OM203341.1, OM203340.1, M203339.1, OM203338.1, OM203337.1, OM203336.1, OM203335.1, OM203334.1, OM203333.1, OM203332.1, OM203331.1, OM203330.1, OM203329.1, OM203328.1, OM203327.1, OM203326.1, OM203325.1, OM203324.1, OM203323.1, OM203322.1, OM203321.1, OM203320.1, OM203319.1, OM203318.1, OM203317.1, OM203316.1, OM203315.1, OM203314.1, OM203313.1, OM203312.1, OM203311.1, OM203310.1, OM203309.1, OM203308.1, OM203307.1, OM203306.1, OM203305.1, OM203304.1, OM203303.1, OM203302.1, OM203301.1, OM203300.1, OM203299.1, OM203298.1, OM203297.1, OM203296.1, OM203295.1, OM203294.1, OM203293.1, OM203292.1, OM203291.1, OM203290.1, OM203289.1, OM203288.1, OM203287.1, OM203286.1, OM203285.1, OM203284.1, OM203283.1, OM203282.1, OM203281.1, OM203280.1, OM203279.1, OM203278.1, OM203277.1, OM203276.1, OM203275.1, OM203274.1, OM203273.1, OM203272.1, OM203271.1, OM203270.1, OM203269.1, OM203268.1, OM203267.1, OM203266.1, OM203265.1, OM203264.1, OM203263.1, OM203261.1, OM203260.1, OM203259.1, OM203258.1, OM203257.1, OM203256.1, OM203255.1, OM203254.1, OM203253.1, OM203252.1, OM203251.1, OM203250.1, OM203249.1, OM203248.1, OM203247.1, OM203246.1, OM203245.1, OM203244.1, OM203243.1, OM203242.1, OM203241.1, OM203240.1, OM203239.1, OM203238.1, OM203237.1, OM203236.1, OM203235.1, OM203234.1, OM203233.1, OM203232.1, OM203231.1, OM203230.1, OM203229.1, OM203228.1, OM203227.1, OM203226.1, OM203225.1, OM203224.1, OM203223.1, OM203222.1, OM203221.1, OM203220.1, OM203219.1, MT859135.1, MH341408.1, MH341407.1, and KX908209.1.

COI gene of *A. mellifera ligustica*: OP763654.1

Full mitochondrial genomes of *A. mellifera carnica*: NC 061380.1, OP404076.1, OP404075.1, MW811175.1 and MN250878.1.

COI gene of *A. mellifera carnica*: OP763654.1, MW082035.1 and MW082034.1.
